# How a single mutation alters the protein structure: a simulation investigation on protein tyrosine phosphatase SHP2†

**DOI:** 10.1039/d2ra07472a

**Published:** 2023-02-01

**Authors:** Yingnan Hou, Xiaoli Lu, Ziyao Xu, Jiarun Qu, Jing Huang

**Affiliations:** a Westlake AI Therapeutics Lab, Westlake Laboratory of Life Sciences and Biomedicine 18 Shilongshan Road Hangzhou 310024 Zhejiang China huangjing@westlake.edu.cn; b Key Laboratory of Structural Biology of Zhejiang Province, School of Life Sciences, Westlake University 18 Shilongshan Road Hangzhou 310024 Zhejiang China; c BioMap 2 Kexueyuan South Road Beijing 100000 China

## Abstract

Protein tyrosine phosphatase SHP2 is a key regulator modulating several signaling pathways. The oncogenic mutation E76K in SHP2 releases the enzyme from an autoinhibited, closed conformation into an active, open conformation. Here, we investigated the conformational dynamics of SHP2 and the effect of the E76K mutation on its conformational ensemble *via* extensive molecular dynamics (MD) and metadynamics (MetaD) simulations. Our simulations provide atomistic details on how the E76K mutated SHP2 prefers the open state and also reveal that the transition between the closed and the open states is highly collective. Several intermediate metastable states during the conformational transition between the closed and the open states were also investigated. Understanding how the single E76K mutation induces the conformational change in SHP2 could facilitate the further design of SHP2 inhibitors.

## Abstract 2

Dissecting how and why a single E76K mutation alters the probability densities of the conformational ensemble of SHP2 with enhanced sampling metadynamics simulations.

## Graphical abstract

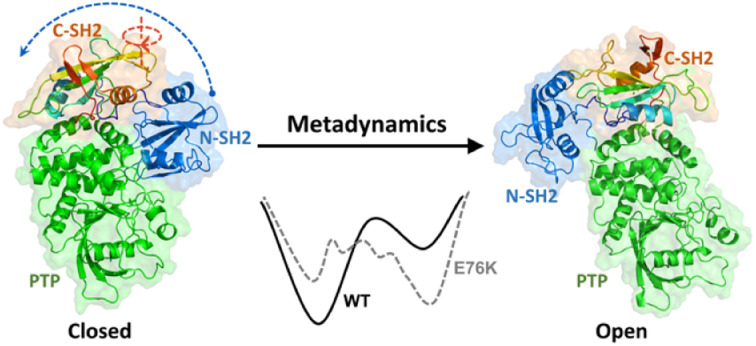

## Introduction

The type 11 non-receptor tyrosine-protein phosphatase SHP2, encoded by the PTPN11 gene, is a critical regulator of signal transduction.^1^ It acts on a variety of downstream receptors and cytoplasmic kinases to regulate cell survival and proliferation primarily through activation of the Ras-Raf-MEK-ERK pathway.^2,3^ It also functions as a key modulator of the programmed cell death 1 (PD-1) and B- and T-lymphocyte attenuator (BTLA) immune checkpoint pathways.^4,5^ SHP2 is an oncogenic tyrosine phosphatase.^6^ Activating mutations of SHP2 are related to developmental disordered diseases such as Noonan syndrome and various cancer types including leukemia, neuroblastoma and lung cancer.^4,7,8^ Targeting SHP2 has been demonstrated to be a successful anti-cancer strategy and small-molecule inhibitors of SHP2 such as TNO155, RMC-4630, and JAB-3068 are under different phases of clinical trials. Recently Choi *et al.* found that the SHP2-MAPK pathway regulates insulin receptor endocytosis such that SHP2 inhibition prolongs insulin action and improves insulin sensitivity, suggesting that SHP2 is also a valuable therapeutic target for diabetes.^9–11^

SHP2 contains two tandemly arranged Src-homology-2 (SH2) domains (N-SH2 and C-SH2), a catalytic protein tyrosine phosphatase (PTP) domain and a C-terminal tail that has at least two phosphatase sites.^12^ The X-ray structure of the wild-type SHP2 (SHP2-WT) reveals that the N-SH2 domain tightly interacts with the PTP domain, blocking the catalytic pocket of the PTP domain being accessible to its substrates and resulting in an autoinhibited closed state (PDB id: 2SHP).^13^ Normally, the autoinhibited conformation of SHP2 is activated by the binding of tyrosine-phosphorylated peptides to the tandem SH2 domains, triggering a series of structural rearrangements in the N-SH2 domain to drive its release from the PTP domain.^14^ Recent investigations on the activation mechanism of SHP2 proposed that the key allosteric switch triggering SHP2 activation might not be the widely-accepted opening of the N-SH2 binding cleft but the opening of the central beta-sheet of N-SH2 based on extensive molecule dynamics (MD) simulations and free energy calculations.^15,16^

**Fig. 1:**
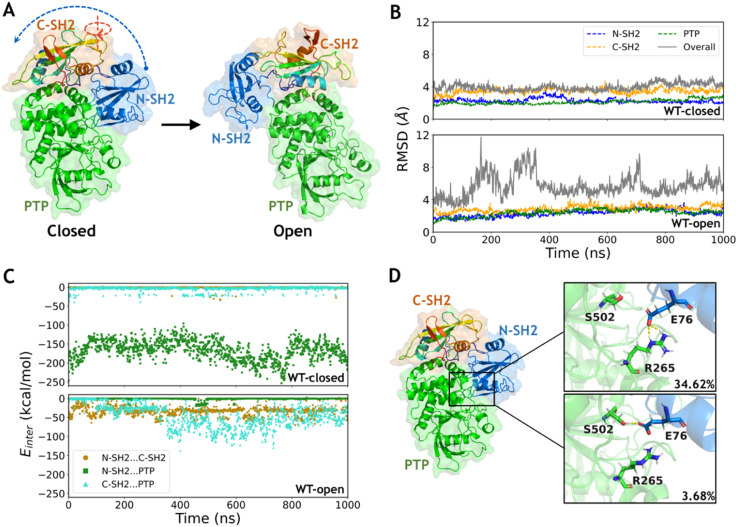
The closed state of wild type SHP2 is more stable than the open state due to the stronger interaction between the N-SH2 and PTP domains. (A) The major structural differences between the closed and open states are the rotation of the C-SH2 domain and the relocation of N-SH2 to form another interface with the PTP surface. (B) The time evolution of RMSDs in each domain and overall structure in the WT-closed and WT-open systems. The RMSDs in each domain were calculated with alignment to that in the first frame (dashed lines), while the overall RMSDs were calculated with alignment to PTP in the first frame (solid lines). (C) The interaction energies between each domain pair during the simulations of the WT-closed and WT-open systems. (D) The hydrogen bond network formed by side chains between the N-SH2 and PTP domains in the WT-closed system with occupancies labeled.

Distinct from the SHP2-WT in the autoinhibited inactivate state, SHP2 with oncogenic mutations shows enhanced basal activity without the stimulation of tyrosine-phosphorylated peptides.^17^ The X-ray structure of SHP2 with the E76K mutation (SHP2-E76K), one of the most frequently observed oncogenic mutations, reveals that the N-SH2 domain locates far away from the catalytic pocket of the PTP domain hence the catalytic site is fully exposed to the solvent (PDB id: 6CRF).^18^ In comparison with the closed conformation of SHP2-WT, there is a dramatic rearrangement of the three domains in SHP2-E76K, including a 120° rotation of the C-SH2 domain with respect to the PTP domain and the relocation of the N-SH2 domain to a totally different interface with the PTP domain (Fig. 1A).^18^ As alternative conformational states of a protein can be beneficially targeted for structure-based rational drug design,^19^ it would be helpful to unravel the conformational ensemble of SHP2.

NMR spectroscopy and X-ray crystallography studies suggested that wild-type SHP2 exchanges between closed and open conformations, while the E76K mutation shifts the equilibrium toward the open conformational state.^20^ Although the dramatic conformational differences between SHP2-WT in the closed conformation and SHP2-E76K in the open conformation can be characterized through their X-ray structures, how the E76K mutation in SHP2 drives such a large conformational change as well as the key points along the transition path are still unclear. It's commonly observed but poorly understood that a single mutation in a protein sequence may alter the structural native state as well as the probability densities of structural ensembles.^21–23^ SHP2 can serve as a model system to study how protein conformational transition can be induced by a single mutation.

In this work, we adopted MD simulations and the enhanced sampling metadynamics (MetaD) to study the conformational dynamics of SHP2 and the effect of the E76K mutation on the conformational transitions. With the X-ray structures of wild type SHP2 at the closed conformation (WT-closed) and E76K mutated SHP2 at the open conformation (E76K-open) determined, we generated the structures of wild type SHP2 at the open conformation (WT-open) and E76K mutated SHP2 at the closed conformation (E76K-closed). From 1 μs conventional MD (cMD) simulations, the dynamic properties of two conformational states in both the WT and E76K systems were investigated. The structural analysis and interaction energy calculations showed that the E76K mutation alters the local conformation and results in the weaker interactions between N-SH2 domain and PTP domain. To further investigate whether and how the single mutation induces the conformational transition from closed state to open state, we defined a series of collective variables (CVs) and employed MetaD to perform the enhanced sampling. Our thermodynamic analysis reveals that E76K mutated SHP2 prefers the open state with high dynamic, while the conformational space for the transition between the two states is high dimensional. Taken together, our dynamic and thermodynamic analysis help to understand how the single mutation E76K alters the conformations of SHP2.

## Results

### The interaction interface between N-SH2 and PTP domains contributes to a highly stable closed state

The two representative states of SHP2 are mainly different in the relative positions among the three domains (Fig. 1A). The conformations of each separated domain are similar in the two crystal structures, in which the root mean square deviation (RMSD) of the backbone heavy atoms (the same below) are 2.1 Å, 1.5 Å and 1.4 Å in the N-SH2, C-SH2 and PTP domains, respectively. However, the RMSD between the overall structures equals 27.8 Å when aligning with the most similar PTP domain, indicating a large conformational change between the closed and the open states. The open conformation of wild type SHP2 is generated by setting the mutation back in the X-ray structure of SHP2-E76K. Conventional MD simulations of SHP2-WT starting from these two states were performed for 1 μs each (ESI, Table S1,† simulation 1–2). To investigate the dynamics of SHP2 in the simulation systems, the time evolution of RMSDs in each domain and the overall structure were calculated with alignment to those in the first frame (the same below). The time evolution of RMSDs in the three domains are 2.3 ± 0.3 Å, 3.5 ± 0.3 Å, 2.1 ± 0.3 Å in the WT-closed system and 2.3 ± 0.4 Å, 2.9 ± 0.4 Å, 2.4 ± 0.4 Å in the WT-open system, respectively (Fig. 1B), indicating the internal stability of the three domains. The overall RMSDs with alignment to the PTP domain are 4.0 ± 0.4 Å in the WT-closed system and 5.7 ± 1.4 Å in the WT-open system, suggesting that the closed state is more stable (Fig. 1B and S1A†). The higher overall RMSDs indicate the relative positions of the three domains are not stable, especially for the open state (Fig. S1B†).

To investigate the contribution of the interaction among the three domains, the inter-domain non-bonded interaction energies were computed from 1 μs MD simulations of the two systems. In the WT-closed system, the interaction energies between the N-SH2 and C-SH2 domains, as well as between C-SH2 and PTP domains are weak (on average −0.7 and −3.7 kcal mol^−1^), while the interaction between N-SH2 and PTP domains is very strong with average interaction energy being −166.5 kcal mol^−1^ (Fig. 1C). In contrast, the interactions between N-SH2 and C-SH2, between C-SH2 and PTP, and between N-SH2 and PTP domains are all relatively weak in the WT-open system, with average interaction energies being −33.2, −46.6 and −1.1 kcal mol^−1^, respectively (Fig. 1C). These results suggest that the interaction interface between the N-SH2 and PTP domain contributes most to stabilizing the closed state.

To further investigate the interaction interface between the N-SH2 and PTP domains, sidechain hydrogen bonding interactions were analyzed. A stable sidechain interaction network is formed in the WT-closed system, including E76 with R265 and with S502 in 34.62% and 3.68% occupancies, respectively (Fig. 1D). In contrast, no hydrogen bond is formed by side chains between the two domains in the WT-open system. These results indicate that the residue E76 does play an important role to maintain the interaction interface between the N-SH2 and PTP domains for stabilizing the closed state for SHP2-WT. Changing the physicochemical properties of this interfacial residue by mutation most likely lead to alteration in conformations. Next, we focused on the effect of conformation change in SHP2 induced by E76K, a single mutation with opposite electrostatic properties.

### E76K mutation drives the escape from the closed state by altering the interaction interface between N-SH2 and PTP domains

**Fig. 2:**
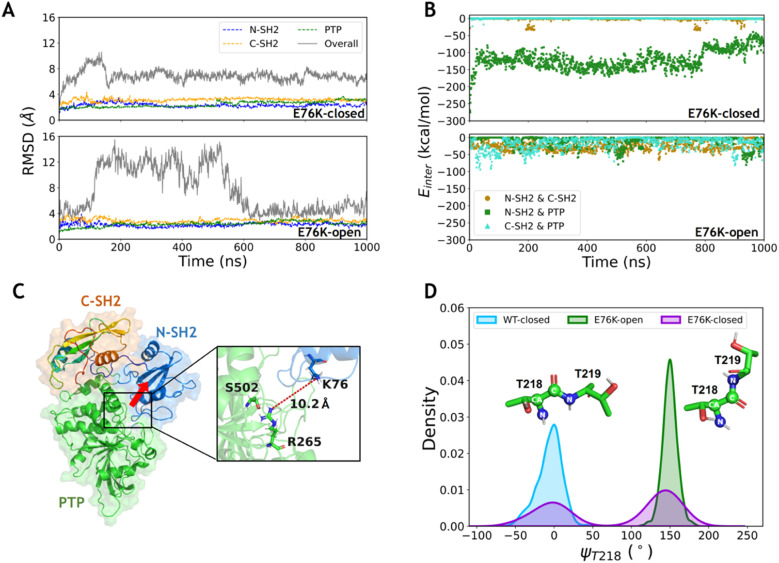
The E76K mutation eliminates the hydrogen bond network between the N-SH2 and PTP domains. (A) The time evolution of RMSDs of each domain and the overall structure for the E76K-closed and E76K-open systems. The RMSDs of each domain were calculated with alignment to that in the first frame (dashed lines), while the RMSDs of the overall structure were calculated with alignment to PTP in the first frame (solid lines). (B) The interaction energies between each domain pair during the MD simulations of the E76K-closed and E76K-open systems. The interaction energy between N-SH2 and PTP domains decreases during the 1000 ns simulations in the E76K-closed systems. (C) N-SH2 detaches from the PTP domain, and no hydrogen bond formed between them. The conformation is the last MD frame for the E76K-closed system, and the red row indicates the direction of the detachment of N-SH2 from the PTP compared with that in the initial closed state. (D) The distributions of the backbone dihedral angle ψ_T218_ in the E76K-closed, E76K-open and WT-closed systems.

To investigate the conformational transition between the closed and open states induced by the typical single mutation E76K in SHP2, MD simulations of SHP2-E76K starting from the closed and the open conformations were carried out for 1 μs each (ESI, Table S1,† simulations 3–4). The time evolution of RMSDs in the three domains (all below 3.2 ± 0.3 Å) are similar with that in the WT systems, indicating that the internal conformations of the three domains are also stable in both the E76K-closed and E76K-open systems (Fig. 2A). However, the overall RMSDs with alignment to the PTP domain are 6.9 ± 0.9 Å in the E76K-closed system and 7.7 ± 3.6 Å in the E76K-open system (Fig. 2A). The observation that both closed and open states are more flexible with the E76K mutation hints an uphill step on the protein fitness landscape.^24^ We note that the melting temperature (*T*_m_) of SHP2 reduces from 51.3 ± 0.1 °C to 42.1 ± 0.1 °C with the E76K mutation.^25^ Structural inspection shows that the relative movements between domains contribute most to the conformational dynamics, especially in the open state (Fig. S1†). Besides, the overall RMSDs in the E76K-open system became small again after ∼600 ns simulation (less than 4.5 Å), suggesting that the open state of SHP2-E76K might be a shallow minimum (Fig. 2A).^18^ The overall RMSDs for the E76K-closed system were significantly larger than those for the WT-closed system, suggesting that E76K mutation might be unfavorable to sustain the closed state.^18,20^

The non-bonded interaction energies among the three domains were calculated from 1 μs MD simulations of the two E76K systems to investigate the contribution of the interaction interface among domains. In the E76K-closed system, the interaction energy between the N-SH2 and PTP domains shows two obvious changes: it rapidly drops from ∼−148.4 kcal mol^−1^ to ∼−129.5 kcal mol^−1^ within the first 50 ns simulation and drops again to ∼−80.5 kcal mol^−1^ after 800 ns (Fig. 2B). This is consistent with the time evolution of the RMSDs of the N-SH2 domain when aligning with the PTP domain (Fig. S1C†), indicating that the relative movement of the N-SH2 domain with respect to the PTP domain leads to the weakening of the interaction energy between them.

Further investigation on sidechain hydrogen bonds between the N-SH2 and the PTP domains finds that no hydrogen bond is formed in the E76K-closed system. The structural analysis for the last frame from 1 μs MD simulation shows that N-SH2 detaches from PTP, resulting the distance between K76 and R265 being larger than 10.2 Å (Fig. 2C). Based on MD simulation results, no new interaction can be formed to compensate the loss of E76-R265 and E76-S502 interactions. Our results indicate that E76K mutation alters the hydrogen bond network in the interaction interface between N-SH2 and PTP domains, thus drives the detachment of N-SH2 from PTP and results in SHP2 escaping from the closed state.

In addition, the backbone dihedral of residue T218 (ψ_T218_) in the linker region between C-SH2 and PTP domains was calculated according to its possible effect on the motion of C-SH2 domain.^20^ Interestingly, the distributions of sampled ψ_T218_ are different in the WT-closed, E76K-open, and E76K-closed systems. The ψ_T218_ was centrally distributed with mean and standard deviation at −5.9 ± 16.3° in the WT-closed system and 149.9 ± 9.5° in the E76K-open system. For the E76K-closed system a bimodal distribution was found with two peaks that correspond to the WT-closed and the E76K-open distributions (Fig. 2D). The transition of this key internal coordinate between the closed and open states was captured during the 1 μs MD simulation in the E76K-closed system. However, the conformational transition from the closed state to the open state is not observed during the 1 μs MD simulation, thus enhanced sampling was adopted to further investigate the conformational changes.

### Conformational transition from the closed to the open state induced by the E76K mutation is highly collective

**Fig. 3:**
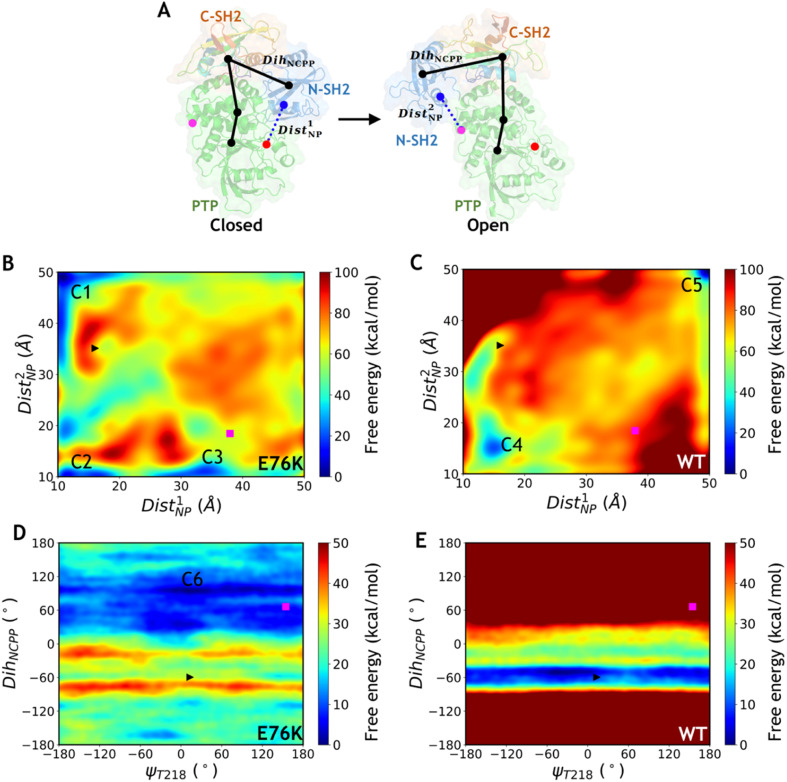
Free energy landscapes obtained by 2D well-tempered MetaD simulations of the E76K and WT systems. (A) Schematic diagram for the definitions of Dist^1^_NP_, Dist^2^_NP_ and Dih_NCPP_. The colored points represent the center of mass of Cα atoms at the interface between N-SH2 and PTP domains. The black points represent the Cα atoms of residues S44, T168, V497 and Q510 for Dih_NCPP_. (B–C) Free energy landscapes projected on the distance CVs Dist^1^_NP_ and Dist^2^_NP_ for the E76K (B) and WT (C) systems. (D–E) Free energy landscapes projected on the dihedral CVs ψ_T218_ and Dih_NCPP_ for the E76K (D) and WT (E) systems. The initial closed state is marked with a black right triangle and the target open state is marked with a magenta square. Some intermediate metastable states are labeled as C1–C6.

Metadynamics is an efficient enhanced sampling technique that biases MD trajectories with a properly chosen set of CVs.^26^ To explore the conformational transition between the closed state and the open state of SHP2 using 2-dimensional (2D) well-tempered metadynamics (WT-MetaD) simulations, we defined several CVs characterizing the structural differences between the two states. Two distances were defined as a pair of CVs using the centers of mass (CoMs) of three groups of Cα atoms selected from rigid regions near the two different interaction interfaces between the N-SH2 and the PTP domains in the two states (Dist^1^_NP_ and Dist^2^_NP_, Fig. 3A, Table 1). In addition, to characterize the global conformational difference, in particular the rotation of the N-SH2 domain with respect to the PTP and the C-SH2 domains, a pseudo dihedral angle was defined by carefully selecting four Cα atoms from the three domains (Dih_NCPP_, Fig. 3A and Table 1), resulting in a difference of about 125° between the two states. Taking Dih_NCPP_ together with the key backbone dihedral ψ_T218_ (Fig. 2D and Table 1), a pair of CVs were constructed to perform MetaD simulations. In addition, the distance between the Cβ atoms of residues K76 and R265 (Dist_K76_R265_, Table 1) combined with Dih_NCPP_ were also used as a pair of CVs for MetaD simulations. We note that all three combinations of paired CVs can distinguish the closed and open states of the crystal structures and conformations from cMD simulations (Fig. S2–S4†). The 2D WT-MetaD simulations for the E76K mutated and the WT systems were carried out starting from the closed state with initial conformations taken from the corresponding 100 ns cMD trajectories (simulation IDs 5–10, Table S1–S2†). Exactly the same biasing parameters such as the height and the width of the Gaussian bias potential were used for the E76K and the WT systems (Table S2†).

**Table 1:** The definitions of the CVs employed in the MetaD simulations together with their values for the crystal structures in the closed and open states

CV	Definition	Closed	Open
Dist^1^_NP_	The distance between the Cα CoM of residues I54-G86 and the Cα CoM of residues Q255-Q257, R465-G467, Q495-V497 and E508-Y511	16.1 Å	37.9 Å
Dist^2^_NP_	The distance between the Cα CoM of residues I54-G86 and the Cα CoM of residues A224-I226, I479-K482 and H520-I522	35.1 Å	18.4 Å
Dih_NCPP_	The dihedral angle among the Cα atoms of S44, T168, V497 and Q510	−59.5°	66.4°
ψ_T218_	The dihedral angle among T218N, T218Cα, T218C and T219N	13.4°	154.5°
Dist_K76_R265_	The distance between atoms K76Cβ and R265Cβ	9.9 Å	47.9 Å

Free energy landscapes projected on the three pairs of CVs for the E76K and WT systems were generated after ∼100 ns MetaD simulations (Table S1–S2†). In all three MetaD simulations deploying different combinations of CVs for the E76K system, the conformational transitions from the closed state to the open state were captured (Fig. 3B and E, S5A†). Driven with the two distance CVs Dist^1^_NP_ and Dist^2^_NP_, the free energy difference between closed and open states (Δ*G*_closed→open_) is −8.2 ± 0.9 kcal mol^−1^ in the E76K system, suggesting that the open state is thermodynamically more stable. In contrast, the closed state is favored in the WT system with a Δ*G*_closed→open_ of 18.4 ± 0.7 kcal mol^−1^ based on the same enhanced sampling setup. While the free energy differences are not quantitatively meaningful due to the difficulties in convergence, it is evident that the E76K mutated SHP2 obtains thermodynamical preference in the open state over the closed state while the opposite holds for the wild type SHP2. These results are consistent with the experimental estimation that the conformations sampling the open states for the E76K mutated and WT SHP2 were 95.8 ± 1.7% and 10.4 ± 1.1% respectively based on NMR chemical shift measurements.^20^

Similar effect of E76K was observed in the MetaD simulations of SHP2-E76K employing the ψ_T218_ dihedral and the Dih_NCPP_ pseudodihedral as paired CVs (Fig. 3D and E). Interestingly, there is almost no free energy barrier along ψ_T218_, which is consistent with transition of ψ_T218_ observed in the microsecond cMD simulations. Starting from the initial closed state (black triangle in Fig. 3), the E76K system underwent conformational change to reach the open state in which Dih_NCPP_ fluctuated around 60°, while the WT system was trapped in the local minimum region of the closed state with Dih_NCPP_ around −60°. Trapping in the closed state local minima was also observed for the MetaD simulations driven with the paired CVs of Dist_K76_R265_ and Dih_NCPP_ paired CVs for the WT system (Fig. S5B†), despite of multiple attempted runs using exactly the same biasing parameters with the E76K system. In summary, MetaD simulations illustrated that the E76K mutation not only modifies the local interactions between N-SH2 and PTP in the closed conformational state but also reshape the overall free energy landscape for SHP2 conformational dynamics. The free energy landscape projected on different CVs are significantly different, which indicates that the conformational transitions between the two states may be highly collective and highlights the difficulties in describing such complicated transitions using only two CVs.

### Multiple intermediate metastable states are captured during the metadynamics simulations

**Fig. 4:**
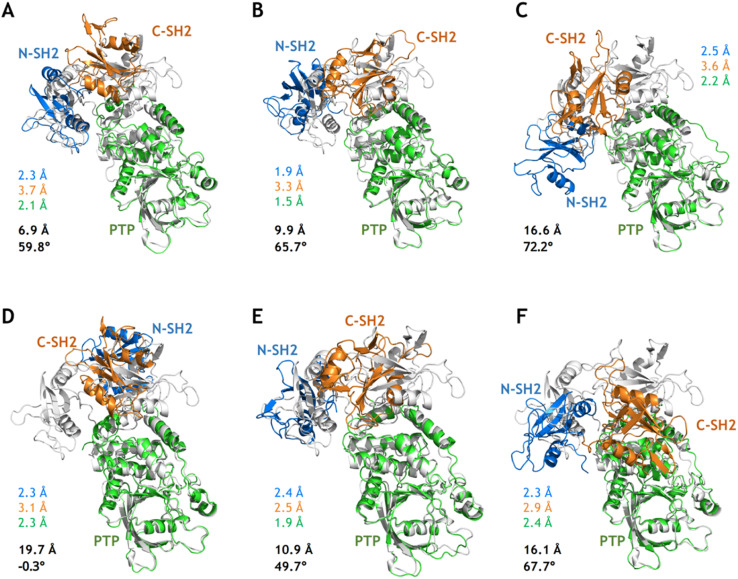
Conformations closest to the target open state from MetaD simulations and after relaxation from cMD simulations. (A–C) The conformations closest to the target open state from the 100 ns MetaD simulations with the distance CVs (Dist^1^_NP_ and Dist^2^_NP_, A), the dihedral CVs (ψ_T218_ and Dih_NCPP_, B) and the distance and dihedral CVs (Dist_K76_R265_and Dih_NCPP_, C), respectively. (D–F) The relaxed conformations after 100 ns cMD simulations for the corresponding conformations in (A–C). The RMSDs in the N-SH2 (blue), C-SH2 (orange), PTP (green) domains with alignment to that in the target open state (light color) are labeled nearby the corresponding conformations. The overall RMSD (black) with alignment to the PTP domain in the target open state and the Dih_NCPP_ are also labeled.

Although MetaD simulations of SHP2-E76K were able to sample conformational states with CVs corresponding to the target open state (the crystal structure of SHP2-E76K), there were still deviations in the atomistic structures as illustrated in Fig. 4A–C. For the conformation closest to the target open state captured from the MetaD simulation employing Dist^1^_NP_ and Dist^2^_NP_ as CVs, the overall backbone RMSD with alignment to the PTP domain in the target open state (the same below) is 6.9 Å and the Dih_NCPP_ equals 59.8° (compared with 66.4° in the target open state, Fig. 4A). For the conformation from MetaD simulation driven with dihedral CVs ψ_T218_ and Dih_NCPP_, the overall RMSD is 9.9 Å and the Dih_NCPP_ equals 65.7° (Fig. 4B). For the conformation from MetaD with the set of CVs Dist_K76_R265_ and Dih_NCPP_, the overall RMSD is 16.6 Å and the Dih_NCPP_ equals 72.2° (Fig. 4C). While MetaD simulations with different sets of CVs can correctly orient the N-SH2 domain with respect to the PTP domain, the precise positioning of the N-SH2 as well as the C-SH2 domains were not yet achieved. The overall RMSD of 6.9 Å for the structure in Fig. 4A can be mainly attributed to the difference in the orientation of C-SH2, and both N-SH2 and C-SH2 were not correctly rotated in the structures in Fig. 4B and C. We also note the deformation of the C-SH2 domain itself, as illustrated by its RMSDs being larger than 3 Å. These results indicate that while the biasing potentials added upon the CVs effectively drove the simulation systems toward the open state, other transition-relevant slow degrees of freedom were not relaxed accordingly.

Starting from the sampled conformations closest to the target open state in the three MetaD simulations, we carried out 100 ns conventional MD simulations (simulation 11–13, Table S1†) and analyzed each of the relaxed conformations from the last MD frames with respect to the target open state. Instead of relaxing into the target open conformational state, all three structures further deviated from it as illustrated by the larger overall RMSDs (Fig. 4). Compared with the unrelaxed conformation from the MetaD employing distance CVs (Fig. 4A), the relaxed conformation after 100 ns cMD simulations adopt larger difference to the target open state with the N-SH2 rotated back and Dih_NCPP_ being −0.3° (Fig. 4D). These results further suggest that the closed-to-open conformational change in SHP2-E76K is a complicated process that enhanced sampling on only two relatively simple CVs might not be sufficient to model.

**Fig. 5:**
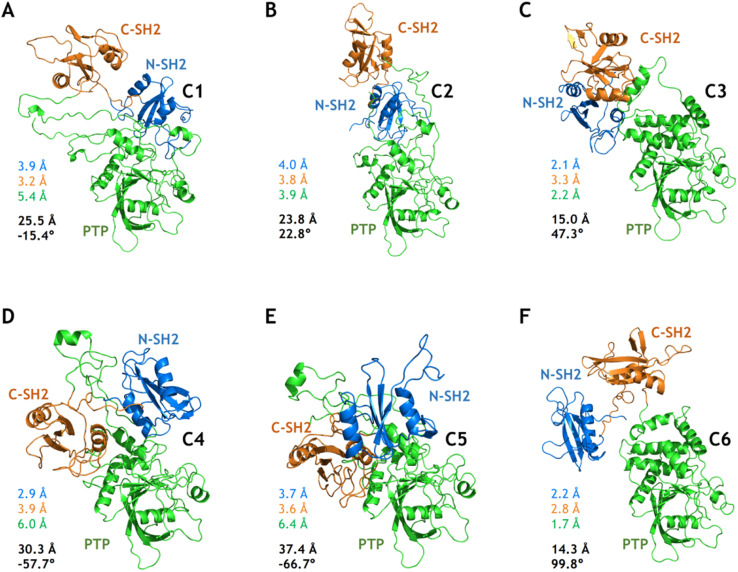
Six intermediate metastable states captured from the MetaD simulations. The conformations in A–F are mapped to the C1–C6 in Fig. 3. The RMSDs in the N-SH2 (blue), C-SH2 (orange), PTP (green) domains with alignment to that in the target open state (light color) are labeled. The overall RMSD (black) with alignment to the PTP domain in the target open state together with the Dih_NCPP_ are also labeled.

Based on the free energy landscapes from the MetaD simulations, a series of intermediate metastable states were sampled (marked C1–C6 in Fig. 3). Each of these intermediate states was inspected with one representative conformation after clustering analyses (Fig. 5). For the intermediate states C1–C3 from the MetaD simulation with the distance CVs (Dist^1^_NP_ and Dist^2^_NP_) for the E76K system, C1 adopts a low Dist^1^_NP_ and a large Dist^2^_NP_, C2 adopts both low Dist^1^_NP_ and Dist^2^_NP_, and C3 adopts a large Dist^1^_NP_ and a low Dist^2^_NP_, respectively (Fig. 3B). The relative positions of the three domains in C1 are more similar to the closed state than the open state, with the overall RMSDs with alignment to PTP in the initial closed and open states being 18.8 Å and 25.5 Å, respectively (Fig. 5A). The three domains, especially the PTP domain, underwent certain degrees of deformation by themselves, with the RMSDs in the N-SH2, C-SH2 and PTP domains when aligning to those in the open state being 3.9 Å, 3.2 Å and 5.4 Å, respectively (Fig. 5A). Besides, the alpha helices in the long linker region between the C-SH2 and the PTP domains (residues N217 to G246) unwind to a loose state (Fig. 5A). For the C2 state, the N-SH2 domain rotated along the PTP domain with the minimal distance of Cα atoms between the two domains being 4.2 Å, resulting in both low Dist^1^_NP_ and Dist^2^_NP_ (Fig. 5B). The C3 conformation represents a metastable state near the target open state, with the overall RMSDs with alignment to PTP in the open state being 15.0 Å and Dih_NCPP_ being 47.3° (Fig. 5C).

For the intermediate states C4–C5 from the MetaD simulation with the distance CVs for the WT system, the relative positions of the three domains are distinct from both the initial closed and the targe open states, with overall RMSD and Dih_NCPP_ are 30.3 Å and −57.7° in the C4 state (Fig. 5D), and 37.4 Å and −66.7° in the C5 state (Fig. 5E), respectively. Deformation of the PTP domain was also observed with the PTP RMSDs being 6.0 Å (C4 state) and 6.4 Å (C5 state). The difference between the C1–C3 metastable states for SHP2-E76K and the C4–C5 states for SHP2-WT was evident. The intermediate state C6 was sampled from the MetaD simulation with the dihedral CVs (ψ_T218_ and Dih_NCPP_) for the E76K system, with overall RMSD being 14.3 Å and Dih_NCPP_ being 99.8° (Fig. 5F). We note that all the six intermediate metastable states sampled from the MetaD simulations were never observed in trajectories of the four 1 μs cMD simulations starting from the closed or the open states.

## Discussion and conclusion

In this work, we investigated the conformational dynamics of SHP2 and the effect of the single E76K mutation on the transition of conformational states using explicit-solvent all-atom simulations starting from two previously determined experimental structures. Microsecond conventional MD simulations indicate that SHP2 in the open state is much more dynamics compared with the autoinhibited closed state in solution, although each domain is stable by itself. Furthermore, well-tempered MetaD simulations provide thermodynamic evidence on that E76K mutated SHP2 prefers the open state and that the transition between the closed and the open states is highly collective.

In particular, our simulations illustrate that both the open and close states of SHP2 exits as basins in SHP2-WT and SHP2-E76K, while the shape of the basins is altered in the free energy landscape as the probability densities of the structural ensembles are changed by the E76K mutation. The conformational transition of multiple conformations can happen at all scales driven by the free energy of the local structures.^27^ The long loop linkers between adjacent domains in SHP2 provide the structural basis for the relatively free movement between domains. In SHP2-E76K, the single mutation significantly reduces the interaction energy between the N-SH2 and the PTP domains, but increases the disorder of the three domains and results in higher conformational flexibility.

Significant advances have been achieved recently in protein structure prediction using end-to-end deep neural networks (DNNs), for example the AlphaFold^28–30^ and the RoseTTAFold.^31^ Further explorations based on AlphaFold have been actively pursued from different aspects, such as sampling alternative conformational states,^32^ establishing protein fold atlas,^33^ and detecting topologically links in predicted structures.^34^ AlphaFold was designed to predict the native structure from coevolution information of a target sequence, where the effect of single mutation was largely filtered during the establishment of coevolution from multiple sequence alignment (MSA). Consequently, AlphaFold shows limited accuracy in predicting the impact of missense mutations as reported by several studies.^35–37^ It is known that one single mutation in a sequence may completely alter the native structure, with additional examples like GA98/GB98 ^21^ and APOE3/APOE4.^38^ It is also common that a single mutation can greatly impact the stability of a protein as shown in mutation-related thermodynamic databases.^39,40^ Actually, understanding the impact of missense mutations on protein structure and dynamics helps to reveal their biological effects as well as roles in related diseases.^36^ Here we dissected the conformational transition induced by a single mutation for the SHP2 system from the physical point of view *via* atomistic simulations. We hope simulation studies could provide helpful insights on the development of DNN-based methods for mutation-sensitive structural prediction.

MetaD is a widely adopted method to extend the sampling capabilities of MD simulations for studying conformational transitions. With the well pre-defined CVs to describe the most relevant (typically also slowest) motion of the system, MetaD simulations have been successfully applied to understand complicated biological processes. For example, using the residual helicity and coordination number as CVs in MetaD simulations, we recently investigated the coupled binding and unwinding process in the α-helical APP TMD recognized by γ-secretase.^41^ In this work, with three sets of manually crafted CVs, we were also able to observe the conformational transition from the close state to the open state in the E76K mutated SHP2 *via* MetaD simulations. However, the free energy landscapes projected on the three sets of CVs are different from each other, and the major conformational states samples by MetaD with different CVs are also significantly different. Both phenomena indicate that the conformational dynamics during the transition might involve a large number of degrees of freedom and be highly collective. The 2D CVs in this study are not sufficient to describe such high dimensional conformational dynamics.

Indeed, one of the main difficulties of bias-based enhanced sampling methods such as MetaD lies in the proper choice of CVs. To maintain the efficiency of exploring the high dimensional conformational space, only a very limited number of CVs can be used, however they need to cover all the slow modes of motion for the simulation system. It is extremely challenging to meet these two conflicting requirements to define proper CVs. In other words, selection of good CVs for MetaD requires to have sufficient prior knowledge of the complicated system, which can often only be gained after successful sampling *via* MetaD simulations. To alleviate the problem of optimal choice of CVs, some strategies have been implemented, including bias-exchange MetaD,^42^ parallel bias MetaD,^43^ and DNN-based CV optimization method such as RAVE.^44^ In addition, we note that CV-independent enhanced sampling methods such as temperature replica exchange or solute scaling might be beneficial for studying such complicated processes.^45,46^

SHP2 is a promising drug target due to its oncogenic role in multiple cancer-critical cellular processes in human cancers.^47^ Efforts to develop small molecules that target SHP2 are ongoing, and several SHP2 allosteric inhibitors are currently in clinical trials for the treatment of solid tumors.^48^ However, while the reported allosteric inhibitors are highly effective against the wild type SHP2, none of them show significant activity against the most frequent oncogenic SHP2 variants including the E76K and the G60R that drive leukemogenesis.^20,48^ In addition, E76K is one of the most observed SHP2 activating mutations in diseases with activation related to the close-to-open conformational change discussed in this work. The small-molecule inhibitor SHP099 has been reported to restore the active form of E76K mutated SHP2, which interacts with the long loop linking the N-SH2 and C-SH2 domains, the loop linking C-SH2 and PTP, as well as the helix of PTP domain.^20^ These loop linkers are found to be important for the flip of the N-SH2 domain and the rotation of the C-SH2 domain in the structural analysis of the multiple key intermediate states from the MetaD simulations. Therefore, our study of the conformational transitions induced by the E76K mutation in SHP2 may provide useful insights into the further drug design targeting SHP2. It would be interesting to simulate the conformational dynamics of SHP2 in the presence of SHP099, which will be pursued in future work.

## Methods

### System setup

The X-ray structure of the wild-type SHP2 (PDB id: 2SHP, chain A and residues K2 to L525) was used to prepare the initial atomic coordinates of the MD simulation systems starting from the closed state. Missing residues were patched based on other available SHP2 structures. CHARMM^49,50^ was used to build the missing hydrogen atom coordinates and perform energy minimization. For the WT-closed system, the SHP2-WT protein was put at the center of a cubic box with a length of 100 Å, which is large enough to contain the protein and at least 16 Å of solvent on all dimensions. It was then solvated with 55 586 explicit TIP3P^51^ water molecules and one sodium ion to neutralize the system, resulting in a system of 175 137 atoms in total. For the E76K-closed system, 55 587 TIP3P water molecules and one chloride ion were added to solvate the SHP2-E76K protein in the closed conformational state, resulting in a total of 175 147 atoms.

Similarly, the crystallographic structure of SHP2-E76K (PDB id: 6CRF, chain A) was used to prepare the initial atomic coordinates for the systems starting from the open state. A cubic box with a length of 100 Å was used. For the WT-open system, SHP2 was solvated with 55 586 TIP3P water molecules and one sodium ion (in total 175 137 atoms). While for the E76K-open system, SHP2-E76K was solvated with 55 587 TIP3P water molecules and one chloride ion (in total 175 147 atoms). Proteins were modeled by the CHARMM36m force field,^52^ which is both accurate and transferable for protein simulations.^53^

### Molecular dynamics and metadynamics simulations

For the four systems, MD simulations were performed using OpenMM^54,55^ with periodic boundary condition (PBC) applied in the isothermal–isobaric (NPT) ensemble. The long-range interactions were evaluated using particle-mesh Ewald (PME) summation with a 12 Å cutoff in real space, with Ewald error tolerance set to 0.001.^56^ The Lennard-Jones interactions were truncated at 12 Å with an atom-based force switching function starting at 10 Å. Hydrogen-containing bonds were constrained and the Langevin integrator was used with a time step of 2 fs. The temperature was maintained at 298 K using the Andersen thermostat^57^ and the pressure was maintained at 1 atm using a Monto Carlo barostat^58^ with the box dimension being updated every 25 steps. Frames were saved every 800 ps.

Metadynamics simulations were carried out using OpenMM with a plugin for PLUMED.^59^ The initial configurations for MetaD simulations were extracted from the corresponding cMD frames (simulation ID: 3) at 100 ns. Simulation details were the same as cMD simulations. The CVs employed in the MetaD simulations were defined referred to the crystal structures in the closed and open states (Table 1). The parameters for applying bias potential in MetaD can be found in the ESI Table S2.†

### Analysis of simulation trajectories

To analysis the sampled structures, VMD^60^ and MDAnalysis^61^ were used to process the trajectories from the MD and MetaD simulations. The structural stability was characterized by RMSD calculations for backbone atoms with different ways of alignment. Hydrogen bonds were counted with a donor–acceptor distance cut-off of 3.5 Å and an angle cut-off of 20°. The non-bonded interaction energies between domains were calculated with CHARMM using the same non-bonded parameters as MD simulations. The free energy profiles were constructed with PLUMED. All structural figures were generated using PyMOL (V2.3.4, http://pymol.org/).

## Conflicts of interest

There are no conflicts to declare.

## Supplementary Material

RA-013-D2RA07472A-s001
