# Supplementary material for: How a single mutation alters the protein structure: a simulation investigation on protein tyrosine phosphatase SHP2
Source: RSC Adv. 2023 Feb 1;13(7):4263–74. doi: 10.1039/d2ra07472a (PMC9891203; doi:10.1039/d2ra07472a)
Supplement: RA-013-D2RA07472A-s001 [file RA-013-D2RA07472A-s001.pdf]

Electronic Supplementary Material (ESI) for RSC Advances.
This journal is © The Royal Society of Chemistry 2023

Supporting Information

How a single mutation alters the protein structure: a simulation investigation on protein tyrosine phosphatase SHP2

Yingnan Hou[1,2], Xiaoli Lu[1,2], Ziyao Xu[3], Jiarun Qu[3], and Jing Huang[1,2 *]

[1] Westlake AI Therapeutics Lab, Westlake Laboratory of Life Sciences and Biomedicine, 18 Shilongshan Road, Hangzhou 310024, Zhejiang, China.

[2] Key Laboratory of Structural Biology of Zhejiang Province, School of Life Sciences, Westlake University, 18 Shilongshan Road, Hangzhou 310024, Zhejiang, China.

[3] BioMap, 2 Kexueyuan South Road, Beijing 100000, China

*Corresponding author: Jing Huang, huangjing@westlake.edu.cn

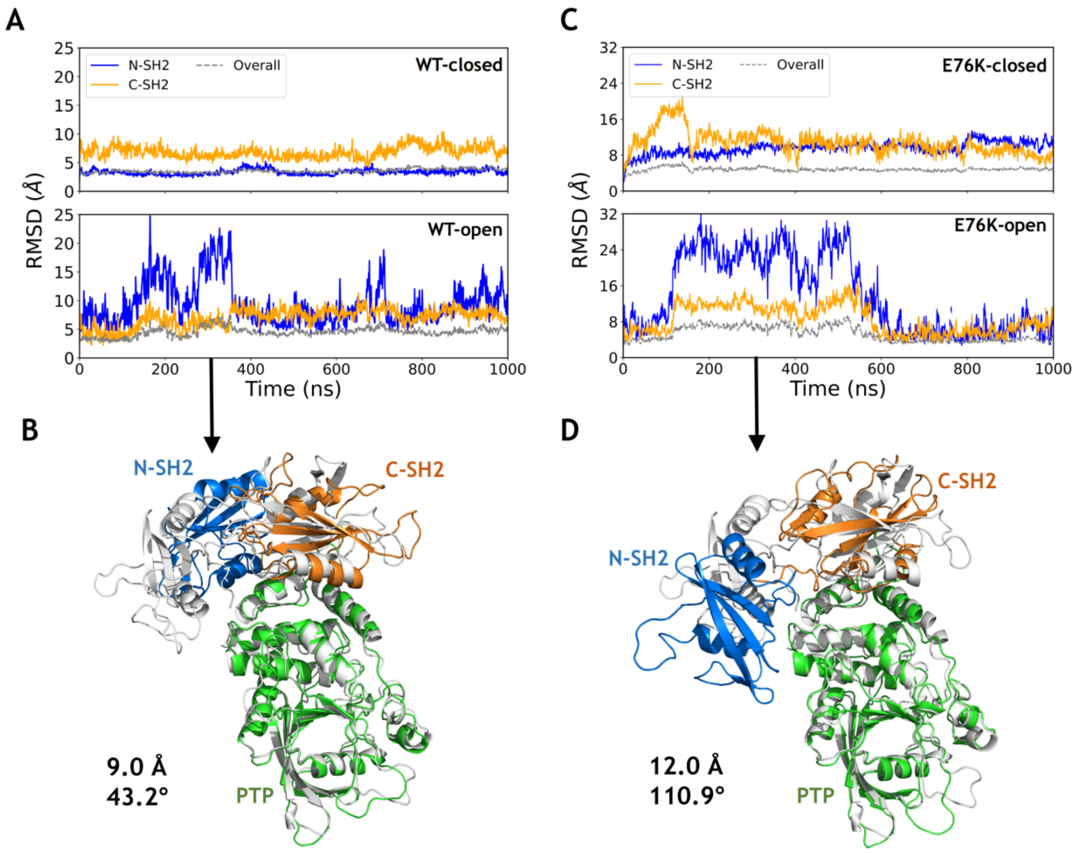

Fig. S1. The RMSDs analysis for the four MD simulations and two representative conformations. (A,C) The time evolution of RMSDs of backbone atoms in the N-SH2 (blue) and C-SH2 (orange) domains with alignment to the PTP domain in the first frame (solid lines). Also shown is the time evolution of RMSDs of backbone atoms in overall structure with alignment to itself in the first frame (dashed gray lines). (B, D) The representative conformations with large RMSDs in the N-SH2 and C-SH2 domains with alignment to the PTP in the first frames, from the MD trajectories in the WT-open (B) and E76K-open (D) systems at ~345 ns. The RMSDs of backbone atoms with alignment to the crystal structure in the open state (light color) and the Dih_{NCPP} are labeled.

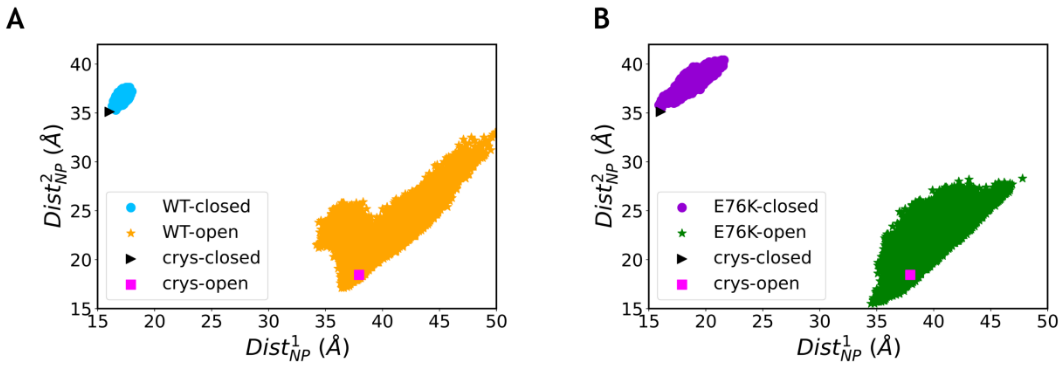

Fig. S2. The conventional MD trajectories in the closed and open states can be well distinguished with the two distance CVs ($Dist^1_{\mathrm{NP}}$ and $Dist^2_{\mathrm{NP}}$). The X-ray structures in the closed (crys-closed) and open (crys-open) states are marked.

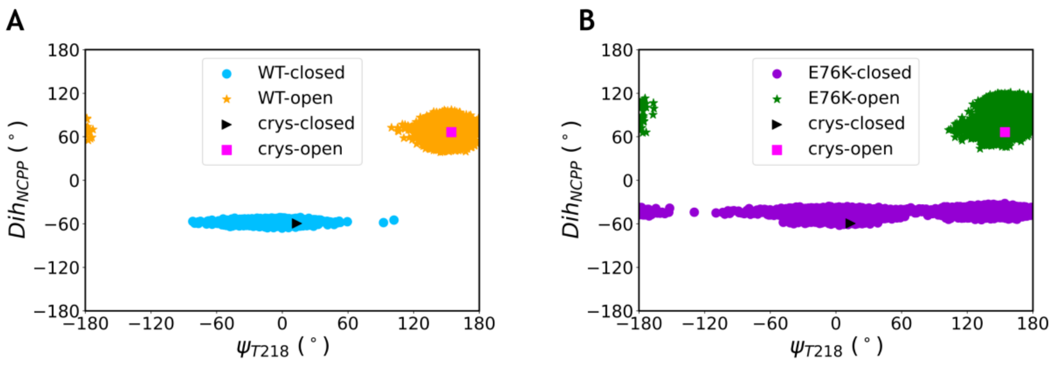

Fig. S3. The conventional MD trajectories in the closed and open states can be well distinguished with the two dihedral CVs (ψ_{T218} and Dih_{NCPP}). The X-ray structures in the closed (crys-closed) and open (crys-open) states are marked.

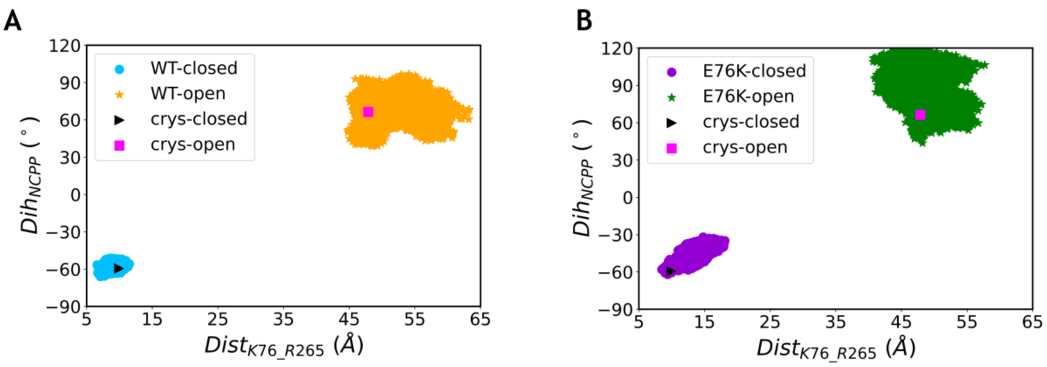

Fig. S4. The conventional MD trajectories in the closed and open states can be well distinguished with the distance $Dist_{\mathrm{K76\_S265}}$ and dihedral Dih_{NCPP}. The X-ray structures in the closed (crys-closed) and open (crys-open) states are marked.

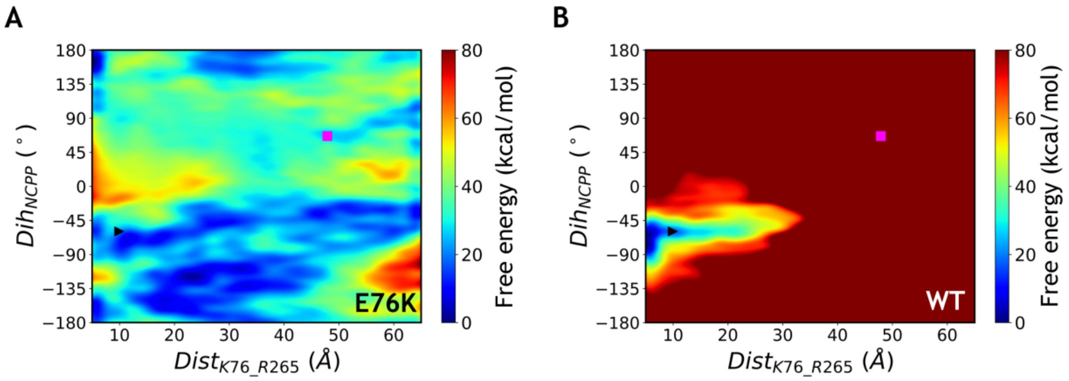

Fig. S5. Free energy landscapes projected on the distance and dihedral CVs for the E76K and the WT systems. The initial closed state is marked with a black right triangle and the target open state is marked with a magenta square.

Table S1. Overall simulations in this work. SHP2-WT: the wild type SHP2; SHP2-E76K: the E76K mutated SHP2. cMD: conventional molecular dynamics simulation; MetaD: Metadynamics simulation; CV: collective variable.

ID	Protein	MD type	Initial conformation	MD Time (ns)
1	SHP2-WT	cMD	2SHP.pdb	1000
2	SHP2-WT	cMD	6CRF.pdb	1000
3	SHP2-E76K	cMD	2SHP.pdb	1000
4	SHP2-E76K	cMD	6CRF.pdb	1000
5	SHP2-E76K	MetaD	After 100ns cMD from simulation 3, distance CV	100
6	SHP2-E76K	MetaD	After 100ns cMD from simulation 3, dihedral CV	100
7	SHP2-E76K	MetaD	After 100ns cMD from simulation 3, distance and dihedral CV	~74.8
8	SHP2-WT	MetaD	After 100ns cMD from simulation 1, distance CV	100
9	SHP2-WT	MetaD	After 100ns cMD from simulation 1, dihedral CV	~33.7
10	SHP2-WT	MetaD	After 100ns cMD from simulation 1, distance and dihedral CV	~9.1
11	SHP2-E76K	cMD	The conformation closest to 6CRF.pdb from simulation 5	100
12	SHP2-E76K	cMD	The conformation closest to 6CRF.pdb from simulation 6	100
13	SHP2-E76K	cMD	The conformation closest to 6CRF.pdb from simulation 7	100

Table S2. Setting for the Metadynamics simulations. Simulation IDs are consistent with that in Table S1. H: HEIGHT, BF: BIASFACTOR, MIN: GRID_MIN, MAX: GRID_MAX, BIN: GRID_BIN, UPP: UPPER_WALLS, LOW: LOW_WALLS. Units for length and energy are nm and kJ/mol. Torsions (dihedral angles) are in radian. NOPBC was used for every atom.

ID	CV1	CV2	SIGMA	H	PACE	BF	TEMP	MIN	MAX	BIN	UPP	LOW	KAPPA
5	$Dist_{NP}^{1}$	$Dist_{NP}^{2}$	0.2,0.2	10	2000	100	298	0,0	8,8	500,500	5,5	1,1	10000
6	ψ_{T218}	Dih_{NCPP}	0.1,0.1	10	2000	100	298	-π,-π	π,π	500,500	/	/	/
7	$Dist_{K76\_S265}$	Dih_{NCPP}	0.2,0.1	10	2000	100	298	0,-π	10,π	500,500	6.5	0.5	10000
8	$Dist_{NP}^{1}$	$Dist_{NP}^{2}$	0.2,0.2	10	2000	100	298	0,0	8,8	500,500	5,5	1,1	10000
9	ψ_{T218}	Dih_{NCPP}	0.1,0.1	10	2000	100	298	-π,-π	π,π	500,500	/	/	/
10	$Dist_{K76\_S265}$	Dih_{NCPP}	0.2,0.1	10	2000	100	298	0,-π	10,π	500,500	6.5	0.5	10000
